# Hermansky-Pudlak Syndrome: A Rare Cause of Post-polypectomy Bleeding

**DOI:** 10.7759/cureus.13781

**Published:** 2021-03-09

**Authors:** Ahmed Baiomi, Hafsa Abbas, Anil Dev

**Affiliations:** 1 Internal Medicine, BronxCare Health System, Bronx, USA; 2 Internal Medicine: Gastroenterology, BronxCare Health System, Bronx, USA; 3 Gastroenterology, BronxCare Health System, Bronx, USA

**Keywords:** hermansky‐pudlak syndrome, post polypectomy bleeding, rectal bleed, gastrointestinal disease, albinism, bleeding diathesis

## Abstract

A colonoscopy is an effective tool for colorectal cancer screening, which aims at identifying precancerous polyps and removing them. Post-polypectomy bleeding (PPB) is one of the most common complications of endoscopic polypectomy. Here, we report a rare and interesting case of a 68-year-old man known to have Hermansky-Pudlak syndrome (HPS) who presented with two days history of rectal bleeding one day after he had a screening colonoscopy with polypectomy. He had a drop in his hemoglobin count and was admitted to the medicine floor and given 1-desamino-8-D-arginine vasopressin (DDAVP). Later, his bleeding stopped and he reported improvement in his symptoms. This case illustrates the importance of considering platelet transfusion and/or administration during minor surgical procedures for patients with bleeding diathesis such as Hermansky-Pudlak syndrome.

## Introduction

Colorectal cancer is the third most common cause of cancer death worldwide [1]. Most cases of colorectal cancer develop through the adenoma-carcinoma cycle. The United States Preventive Services Task Force recommends colorectal cancer screening for asymptomatic individuals above the age of 50 years [2]. The aim of screening colonoscopy is to identify and remove adenomatous polyps that may progress to colon cancer. Post-polypectomy bleeding is a known complication after colonoscopy. Studies have reported the rate of post-polypectomy bleeding as 0.4% per colonoscopy performed [3]; here, we report an interesting case of post-polypectomy bleeding in a patient with Hermansky-Pudlak syndrome (HPS).

## Case presentation

A 68-year-old Hispanic man of Puerto Rican descent presented to the emergency department with a two-day history of a small amount of bright red rectal bleeding twice daily. He denied any associated symptoms like abdominal pain, nausea, vomiting, diarrhea, or constipation, shortness of breath, dizziness, or syncope. He was noted to have thrombocytopenia with a platelet count of 87 k/ul. One day prior to this event, he underwent a screening colonoscopy that showed two polyps in the ascending colon (12 and 14 mm in size) removed with hot snare polypectomy. Post-intervention hemostatic clip and epinephrine were injected. Pathology showed inflammatory polyps. He has a background history of hypertension, dyslipidemia, coronary artery disease, Hermansky Pudlak syndrome, squamous cell cancer of the skin status post-irradiation therapy eight years ago. On initial examination in the emergency department, the patient was afebrile, with stable vital signs. His abdominal examination revealed no tenderness and the rectal exam showed brown stool. The rest of the review of systems and physical examination was unremarkable.

Laboratory tests showed a decrease in his hemoglobin from a baseline of 13 gm/dl to 11.0 gm/dl and thrombocytopenia to 80k/ul, the rest of his laboratory workup was normal. Hematology service was consulted and advised to give 20 mg of 1-desamino-8-D-arginine vasopressin (DDAVP). The patient was admitted to the medicine floor for observation. He did not have any further episodes of rectal bleeding, his hemoglobin level remained stable, and he was discharged with gastroenterology follow-up.

Later, he was seen after discharge in the gastroenterology clinic, where he reported that he was symptom-free and reported no further episodes of rectal bleeding.

## Discussion

Hermansky-Pudlak syndrome is a rare autosomal disorder characterized by oculocutaneous albinism, a bleeding diathesis secondary to platelet dysfunction, and other organ involvement specific to certain types, including granulomatous colitis and pulmonary fibrosis [4]. Hermansky-Pudlak syndrome results from the abnormal formation of intracellular vesicles resulting in melanosome dysfunction leading to oculocutaneous albinism and the absence of platelet-dense bodies leading to bleeding diathesis. Mistrafficking leads to pulmonary fibrosis and granulomatous colitis [5]. Ten subtypes of Hermansky-Pudlak syndrome have been identified depending on the defective gene leading to different presentations [6].

Hermansky-Pudlak syndrome is a rare disease with a prevalence of 1 in a million. However, it is common in Puerto Ricans, affecting approximately one in 1,800 persons, and approximately one in 22 are carriers of the gene [7].

The clinical presentation of Hermansky-Pudlak syndrome may vary among the 10 subtypes depending on the defective gene, however, all subtypes are characterized by oculocutaneous albinism and platelet storage pool deficiency. HPS-1 and HPS-4 are recognized as severe forms of the disorder and they usually have granulomatous colitis and pulmonary fibrosis. HPS-3 patients can have colitis but have no manifested pulmonary fibrosis. Types 3, 4, and 5 are associated with a milder presentation.

Cutaneous manifestation includes a decrease in skin and hair pigmentation varying from a complete absence of color to a relative decrease in skin and hair color. Ocular manifestations include absence or decrease in retinal pigment and macular degeneration resulting in photophobia, decreased visual acuity, and nystagmus [8]. Bleeding diathesis due to platelet dysfunction will result in easy bruising, gingival bleeding, and prolonged bleeding after minor procedures, including circumcision, dental extraction, and normal vaginal delivery [9]. Pulmonary fibrosis will result in progressive dyspnea on exertion and respiratory failure [10]. Granulomatous colitis will present with symptoms like those of inflammatory bowel disease like abdominal pain, fever, weight loss, malabsorption, and frequent watery and bloody diarrhea [11].

The diagnosis of Hermansky-Pudlak syndrome is usually suspected when a patient with diffuse hypopigmentation of the skin, hair, iris, and retina presents with bleeding diathesis especially if he reports Puerto Rican ancestry. Routine blood tests will show prolonged bleeding time with normal prothrombin time and partial thromboplastin time, but patients would have abnormal platelet aggregation using platelet stimulants such as adenosine diphosphate or epinephrine, and a platelet aggregometer. Electron microscopy will show the absence of dense granules, which are essential for hemostasis and their absence will result in bleeding diathesis [12]. Chest X-ray and computed tomography (CT) can show increased reticular opacities, thickened interlobular septa, and ground-glass infiltrates in addition to fibrotic changes, including traction bronchiectasis and honeycombing in patients with subtypes leading to pulmonary fibrosis [13]. A colonoscopy will show superficial crypt abscesses, a prominent inflammatory cell infiltration in involved areas, and inflammatory polyps in patients with granulomatous colitis. The definitive diagnosis is through genetic testing, which will also help in the identification of the subtype through the involved defective gene.

There is no definitive treatment for Hermansky-Pudlak syndrome. Our goal is to help the patient manage the symptoms that may arise and in secondary prevention. The patient should wear protective sunglasses and avoid exposure to the sun to protect against the development of skin cancers. Bleeding episodes can be handled with local pressure when applicable or platelet transfusion and the use of desmopressin. Also, surgery should be planned in coordination with a hematology consultation, as DDAVP and platelet transfusions may be needed [11,14].

## Conclusions

Colorectal cancer can be prevented through a colonoscopy with the removal of precancerous polyps. Hermansky-Pudlak syndrome is a syndrome characterized by oculocutaneous albinism and bleeding diathesis due to platelet aggregation defect, which could result in increased risk for bleeding in both major and minor ambulatory procedures. Careful planning and consideration of the elevated risk of post-procedure bleeding in these patients is recommended.
